# Epigenetic MMR defect identifies a risk group not accounted for through traditional risk stratification algorithms in endometrial cancer

**DOI:** 10.3389/fonc.2023.1147657

**Published:** 2023-04-06

**Authors:** Courtney J. Riedinger, Morgan Brown, Paulina J. Haight, Floor J. Backes, David E. Cohn, Paul J. Goodfellow, Casey M. Cosgrove

**Affiliations:** ^1^ Department of Obstetrics and Gynecology, Division of Gynecologic Oncology, Arthur G. James Cancer Hospital, The Ohio State University Wexner Medical Center, Columbus, OH, United States; ^2^ Department of Obstetrics and Gynecology, The Ohio State University Wexner Medical Center, Columbus, OH, United States

**Keywords:** mismatch repair deficiency (MMR), epigenetic loss, Lynch syndrome, biomarker, risk stratification, endometrial cancer

## Abstract

**Purpose:**

We sought to evaluate the contribution of mismatch repair (MMR) status to traditional risk stratification algorithms used to predict nodal involvement and recurrence in a large single-institution cohort.

**Methods:**

Endometrioid endometrial cancer (EC) cases from 2014-2020 were evaluated. MMR immunohistochemistry (IHC) was performed universally. Uterine factors assessed in the Mayo criteria were used to retrospectively classify patients as low or high risk for lymphatic spread. Patients were classified according to risk for recurrence using GOG 99 and PORTEC criteria. Associations were evaluated using chi-square and t-tests and contributing factors assessed using logistic regression models.

**Results:**

1,514 endometrioid EC were evaluated; 392 (25.9%) were MMR (MMR) deficient of which 80.4% of MMR defects were associated with epigenetic silencing of *MLH1*. Epigenetic MMR defects were significantly more likely to be high risk for lymph node (LN) metastasis based on Mayo criteria (74.9% vs 60.6%, *p*=<0.001) and with the presence of LN metastasis (20.3 vs 10.5%, *p*=0.003) compared to MMR proficient tumors. Tumors with epigenetic MMR defects were significantly more likely to be classified as high or high intermediate risk using GOG99 and PORTEC criteria. Furthermore, cases with epigenetic MMR defects classified as low or low intermediate risk were significantly more likely to recur (GOG99 *p*=0.013; PORTEC *p*=0.008) and independently associated with worse disease-free survival (DFS). MMR status was found to be independently associated with worse DFS (HR 1.90; 95% CI 1.34-2.70; *p*=0.003) but not overall survival.

**Conclusion:**

While MMR deficient EC has been associated with poor prognostic features in prior reports; we demonstrate that only epigenetic MMR defects have poorer outcomes. Epigenetic MMR defect were independently associated with lymph node metastasis after controlling for risk criteria. Epigenetic MMR deficiency was found to be an independent predictor of recurrence beyond the factors considered in traditional risk stratification algorithms. Traditional uterine-based risk stratification algorithms may not fully reflect the risk for recurrence in MMR deficient tumors. Consideration should be given to implementing MMR status and *MLH1* hypermethylation alongside traditional risk stratification algorithms. Performing MMR IHC on preoperative pathologic specimens may aid in risk stratification and patient counseling.

## Introduction

Endometrial cancer (EC) is the most common gynecologic malignancy in the U.S. and more than 66,000 new cases will be diagnosed in 2023. MMR (MMR) deficiency is common in EC, occurring in 20-40% of cases (1, 2). Determination of MMR status in EC has several clinical implications. Loss of expression of MMR proteins may be associated with inherited germline defects in MMR genes (*MLH1, MSH2, MSH6, PMS2*). Approximately 3-5% of EC may be attributed to Lynch Syndrome (LS), a hereditary cancer predisposition caused by mutations in mismatch repair (MMR) genes. Women with LS have up to a 60% lifetime risk for developing EC as well as a significant risk for colorectal, ovarian, stomach, and other cancers (3–6). EC serves as an important ‘sentinel’ cancer and is the first cancer diagnosed in approximately 50% of women with LS (6, 7). While MMR deficiency in EC is common, the majority of cases can be explained by epigenetic silencing of the *MLH1* promoter rather than germline defects (1, 2, 8, 9). While *BRAF^V600E^* mutations are frequently implicated in sporadic colorectal cancer (10), *BRAF* mutations are very rare in EC (0.1%) and testing is not recommended as part of universal screening for LS (11, 12).

Outside of genetic screening, MMR status is an important prognostic biomarker (13–15) and can be used to predict response to immunotherapy (16, 17). Currently, MMR testing is recommended by the National Comprehensive Cancer Network (NCCN) as a complement to morphologic assessment of EC and is used to separate EC into one of four molecular subgroups (*POLE* mutated, MMR-deficient/Microsatellite instability-high, copy number low, and copy number high) (18, 19). Our group, and others, have reported on the association between MMR deficiency and a number of poor prognostic indicators routinely used to guide the decision for adjuvant therapy in endometrioid EC. Epigenetic MMR defects have been associated with diagnosis at an older age, the presence of lymphovascular space invasion (LVSI), and higher-grade tumors, as well as diagnosis at a more advanced stage. EC with *MLH1* hypermethylation has also been associated with larger tumor volumes increasing the risk for lymph node metastasis (9, 20). However, even with these poor prognostic features, data regarding outcomes in EC with MMR deficiency and epigenetic MMR defects have been inconsistent (9, 21). While many groups have reported on reduced recurrence free survival in EC with MMR defects others have reported that there is no effect or even an improvement in OS in these tumors (11, 22–33).

We sought to determine if MMR status might add to traditional risk stratification algorithms used to predict risk for lymph node metastasis and recurrence in a large, single-institution cohort.

## Materials and methods

This was an institutional review board-approved retrospective review from the Ohio State University Comprehensive Cancer Center (OSUCCC) from June 1, 2014 to December 31, 2020. All patients who underwent surgery for an EC diagnosis at our institution were included. Clinical and demographic data were abstracted from medical records. Electronic health information exchange (HIE) was used to access medical records from outside institutions where available. A portion of this cohort was included in previous reports (20, 22).

Universal MMR IHC testing for protein expression of MLH1, PMS2, MSH2, and MSH6 was performed clinically on all EC specimens for LS screening as standard of care. Tumors with loss of expression of MLH1 or PMS2 on IHC underwent reflex *MLH1* methylation testing using methylation-specific PCR to triage for genetics referral. MMR status of tumors was classified as MMR proficient (normal) if there was intact expression of MMR proteins. Patients’ tumors with loss of MLH1/PMS2 on IHC and methylation of the *MLH1* promoter region were classified as having an epigenetic MMR defect. Tumors with abnormal IHC without *MLH1* methylation were classified as MMR deficient due to a probable MMR mutation (probable Lynch syndrome or double somatic mutation).

The criteria established by Mariani et al. from the Mayo Clinic (i.e. tumor diameter, grade, and depth of invasion) were used to retrospectively classify patients as low or high risk for lymphatic spread (23). Patients were classified as low risk for lymph node metastasis if they were without evidence of extrauterine disease, with primary tumor diameter ≤2cm, FIGO grade 1 or 2 histology, and ≤50% myometrial invasion. Tumor grade and depth of myometrial invasion were abstracted from the final pathology report. Tumor size was based on hysterectomy gross tumor specimen measurements recorded by the evaluating pathologist. Tumor volume was calculated using the maximum tumor measurements for 3 lengths as previously described (20). Subjects were classified according to GOG99 and PORTEC risk criteria as previously reported (34–36). Briefly, patients were classified as high intermediate risk (HIR) by GOG 99 depending on age and the number of risk factors (grade 2 or 3 tumor, the presence of LVSI, and outer 1/3 myometrial invasion). Patients were classified as high intermediate risk by PORTEC if they had 2 of 3 clinicopathologic factors: age > 60 years, ≥50% myometrial invasion, and grade 3 histology.

Clinical-pathologic relationships were assessed using χ2, Fisher’s exact test, and t test. Where data were not normally distributed, the Wilcoxon signed-rank test was utilized. The Kaplan-Meier product limit was used to estimate survival. The log-rank test was used to test for differences in survival. Multivariable logistic regression models were developed, and odds ratios (ORs) were used to evaluate the risk factors associated with recurrence. Cox proportional hazard models were used to assess variables associated with disease-free (DFS) and overall survival (OS). All statistical analyses were performed using JMP^®^ Pro, Version 15.2.0. SAS Institute Inc., Cary, NC, 2019.

## Results

Data was collected for 1,718 ECs; for the purposes of this study analyses were limited to endometrioid histology EC (N=1,514). The median follow-up time was 2.5 years (range 20 days to 7.8 years). The clinical and pathologic features of the entire cohort, stratified by MMR status are presented in **Table 1**. Most patients were obese (82%), were stage I at diagnosis (83%), and had grade I tumors (81%). Three-hundred ninety-two (25.9%) patients’ tumors demonstrated MMR defect based on IHC. Eighty percent (315/392) of those were associated with *MLH1* hypermethylation and classified as epigenetic MMR defects. Seventy-seven patients had MMR IHC loss of expression without *MLH1* hypermethylation suggestive of MMR mutations. IHC staining for these cases revealed 15 with loss of MLH1/PMS2 without MLH1 hypermethylation, 9 with isolated loss of PMS2 without MLH1 hypermethylation, 25 with loss of MSH2/MSH6, and 28 with isolated loss of MSH6 staining. Of those 77 patients, germline testing results were available in 53 cases, 38 of whom (2.5% of the entire cohort) had confirmed LS. *MSH6*-related LS was diagnosed in 15 cases, *PMS2*-related LS in 14 cases, *MSH2*-related LS in 7 cases, and *MLH1*-related LS in 2 cases.

**Table 1:** Clinical-pathologic features of Endometrioid EC by MMR status.

Clinical-pathologic features	MMR proficient	Epigenetic loss *MLH1*	Probable MMR mutation	*p* value
	*N* = 1122 (%)	*N* = 315 (%)	*N* = 77 (%)	
Age				
Mean (SD)	59.6 (11.16)	64.67 (9.55)	55.08 (9.23)	**≤**0.001
Median (Range)	60.0 (25-94)	64.0 (35-90)	56.0 (37-76)	
BMI				
Mean (SD)	39.08 (10.61)	37.3 (8.65)	34.2 (9.69)	≤0.001
Median (Range)	38.28 (18.6-81.3)	36.4 (19.4-66.4)	32.2 (20.4-62.4)	
Racial/Ethnic Group				0.240
White	1060 (94.5)	296 (93.8)	71 (92.2)	
Black	34 (3.0)	14 (4.4)	2 (2.6)	
Asian	12 (1.1)	4 (1.3)	4 (5.2)	
Other	16 (1.4)	1 (0.3)	0	
Stage				≤0.001
I	988 (88.1)	229 (72.7)	66 (85.7)	
II	31 (2.8)	11 (3.5)	2 (2.6)	
III	86 (7.7)	57 (18.1)	7 (9.1)	
IV	17 (1.5)	18 (5.7)	2 (2.6)	
FIGO Grade				≤0.001
1	962 (85.7)	199 (63.2)	65 (84.4)	
2	116 (10.3)	77 (24.4)	6 (7.8)	
3	44 (3.9)	39 (12.4)	6 (7.8)	
LVSI				
Present	164 (14.6)	118 (37.5)	12 (15.6)	≤0.001
Absent	958 (85.4)	197 (62.5)	65 (84.4)	
Mayo criteria				
High risk	690 (61.5)	249 (79.0)	48 (62.3)	**≤**0.001
Low risk	432 (38.5)	66 (21.0)	29 (37.7)	
GOG 99 risk classification				
Low risk	292 (26.0)	40 (12.7)	19 (24.7)	≤0.001
Low intermediate risk	621 (55.4)	133 (42.2)	46 (59.7)	
High intermediate risk	104 (9.3)	67 (21.3)	3 (3.9)	
High risk	105 (9.4)	75 (23.8)	9 (11.7)	
PORTEC risk classification				
Low risk	832 (74.3)	175 (55.6)	58 (75.3)	**≤**0.001
High intermediate risk	146 (13.0)	43 (13.7)	7 (9.1)	
High risk	142 (12.7)	97 (30.8)	12 (15.6)	
Adjuvant therapy				
Chemotherapy	60 (5.4)	29 (9.2)	7 (9.1)	≤0.001
Chemo + radiation	69 (6.2)	49 (15.6)	6 (7.8)	
Radiation	105 (9.4)	50 (15.9)	9 (11.7)	
Other	6 (0.5)	2 (0.6)	0	
None	882 (78.6)	185 (58.7)	55 (71.4)	
Recurrence/Progression				
Yes	43 (3.8)	48 (15.2)	3 (3.9)	**≤**0.001
No	1079 (96.2)	267 (84.8)	74 (96.1)	

The only significant difference between patients with a probable MMR deficiency and those who were MMR proficient was age (median 56 vs 60 years, *p*=0.009) and BMI (median 32.2 vs 38.3, *p*=0.003). EC with probable MMR deficiency did not differ from MMR proficient EC in terms of stage, grade, LVSI, the receipt of adjuvant therapy, Mayo risk criteria, GOG 99, or PORTEC risk criteria. Comparatively, ECs with epigenetic loss of *MLH1* were significantly more likely to be diagnosed at a more advanced stage (23.8%), with higher grade tumors (12.4%), with LVSI (37.5%), and to receive adjuvant therapy (41.2%) (**Table 1**).

## MMR status and traditional risk stratification algorithms

### Mayo criteria

Given the reported increased risk of lymph node (LN) metastasis in EC with epigenetic loss of *MLH1* we evaluated the contribution of MMR status to Mayo criteria. Mayo criteria published by Mariani et al. has been used to identify patients which may safely be excluded from routine lymphadenectomy due to low risk of LN metastasis (37). We evaluated 1,477 endometrioid EC without preoperative evidence of advanced disease (without evidence of metastatic disease or lymphadenopathy on preoperative imaging). The majority of EC in our cohort (77.8%) underwent LN assessment (sentinel lymph node biopsy or full lymphadenectomy) regardless of MAYO criteria risk. MMR deficient EC were significantly more likely to be deemed high-risk for lymph node metastasis by Mayo criteria (74.9% vs 60.6%, *p*=**≤**0.001) (**Table 1**). However, there was no significant difference in the characteristics that resulted in exclusion from the low-risk group (ie. Tumor size, myometrial invasion, grade) between MMR deficient and MMR proficient EC. In addition, in patients at high risk for lymphatic spread by Mayo criteria, ECs with epigenetic MMR defect were significantly more likely to have LN metastasis (20.3% vs 10.5%, *p*=0.003). There was no significant difference in the rate of LN metastasis between patients with probable MMR mutation and MMR proficient EC after selecting for those at high-risk by Mayo criteria (15.4% vs 10.5%, *p*=0.369). While Mayo criteria is not used routinely to omit lymph node assessment in our practice due to high utilization of sentinel lymphadenectomy, there was a significantly higher rate of lymphadenectomy in patients with MMR deficient EC compared to MMR proficient EC (83.7% vs 74.1%, *p*=**≤**0.001) reflecting the impact that intraoperative assessment of tumor volume may have in surgical decision making. Sixty-seven percent of patients at low risk by Mayo criteria underwent surgical lymph node assessment. There were 4 cases of lymph node metastases in patients deemed low risk by Mayo criteria; two of these occurred in patients with epigenetic MMR defect, and two in MMR proficient EC. The false negative rate of Mayo criteria in epigenetic MMR defects was 3.9% (compared to 0.7% in MMR proficient) (HR 5.44, 95% CI 0.78-37.8, *p*=0.105). There were two retroperitoneal recurrences that could be related to undiagnosed lymphatic spread in patients who did not undergo lymph node assessment; both patients were high-risk by Mayo criteria but did not undergo lymphatic dissection due to inadequate visualization and medical comorbidities. Both patients had MMR deficient tumors (one epigenetic loss and one with a probable MMR mutation). A nominal logistic regression model was used to evaluate the risk for lymph node metastasis. Epigenetic MMR defect was found to be an independent risk factor for lymph node metastasis (HR 2.52; 95% CI 1.65-3.85; *p*=**≤**0.001) after controlling for risk group by Mayo criteria, LVSI, and tumor volume (**Table 2**).

**Table 2:** Multivariate analysis risk for LN metastasis and risk for recurrence in endometrioid EC.

Variable	Risk for Lymph Node Metastasis			Risk for Recurrence		
	Hazard ratio	95% CI	*p* value	Hazard ratio	95% CI	*p* value
Mayo criteria (high risk vs low risk)	5.60	4.62-24.78	**≤**0.001*			
Tumor volume (continuous variable)	1.40	1.21-1.62	0.003*	0.99	0.99-1.01	0.153
LVSI (present vs absent)	6.30	2.81-18.67	**≤**0.001*	2.37	1.34-4.19	0.003*
MMR status (MMR deficient vs MMR proficient)	1.99	1.31-3.03	0.001*	2.34	1.25-4.39	0.008*
MMR status (epigenetic vs MMR proficient)	2.52	1.65-3.85	**≤**0.001*	2.74	1.44-5.25	0.002*
MMR status (probable MMR mutation vs MMR normal)	1.29	0.49-3.44	0.600	0.66	0.09-4.95	0.683
Age (continuous variable)	0.50	0.12-2.02	0.333	1.03	0.94-1.35	0.238
BMI (continuous variable)	0.99	0.97-1.02	0.532	0.99	0.96-1.03	0.747
FIGO Grade (grade 1 & 2 vs 3)	0.38	0.21-0.68	0.001*	0.4	0.22-0.73	0.0027*
Stage (III/IV vs I/II)				3.68	2.08-6.54	**≤**0.001*
Adjuvant therapy (therapy vs no therapy)				0.67	0.19-0.37	0.193
GOG99 (HIR vs LIR or Low risk)ª				3.94	2.09-7.44	**≤**0.001*
PORTEC (High risk or HIR vs Low risk)ª				3.62	1.98-6.61	**≤**0.001*

*Denotes statistical significance. Data from N=1,514 endometrioid endometrial cancers except for ª which evaluates 1,327 stage I and II endometrial cancers. N=1,178 EC underwent lymph node dissection, N=112 EC with lymph node metastasis. N=97 with recurrent disease.

LN, (Lymph Node); LVSI, (Lymphovascular space invasion); MMR, (Mismatch Repair); HIR, (High intermediate risk); LIR, (Low intermediate risk).

### GOG99 and PORTEC risk classification

Adjuvant radiation has not been shown to improve survival in early-stage disease (34–36) and the role of adjuvant therapy in early-stage endometrial cancer remains uncertain. The GOG99 and PORTEC studies evaluated the role of adjuvant therapy in early-stage endometrial cancer (34–36) and identified patients that would benefit from adjuvant radiation to decrease the risk for pelvic recurrence. These trials arrived at different (but overlapping) criteria to determine high intermediate risk. We categorized the 1,327 early-stage endometrioid EC in our cohort according to the GOG99 and PORTEC criteria: Eighty seven percent were deemed low or low intermediate risk by GOG99 criteria, and 80.9% low risk by PORTEC criteria. MMR deficient ECs were significantly more likely to meet GOG99 HIR criteria (23.1% vs 10.3%, *p*=**≤**0.001) and high risk or HIR by PORTEC (23.4% vs 17.8%, *p*=0.004) (**Table 1**). A nominal logistic regression model was used to evaluate the risk of recurrence after controlling for risk classification. After controlling for GOG99 classification and PORTEC classification, MMR status as a dichotomous variable was found to be an independent risk factor for recurrence, (HR 2.34; 95% CI 1.25-4.39; *p*=0.008). When MMR status was evaluated as a trichotomous variable only epigenetic loss (rather than probable MMR mutation) remained independently associated with recurrence (HR 2.74; 95% CI 1.44-5.25; *p*=0.002) after controlling for GOG99 and PORTEC classification (**Table 2**). Epigenetic loss of *MLH1* also demonstrated significant association with recurrence after correcting for receipt of any adjuvant therapy and type of adjuvant therapy. Indeed, EC with epigenetic MMR defect had a statistically and clinically meaningful increased rate of recurrence in early-stage EC compared to MMR proficient EC (7.9% vs 2.4%, *p*=0.005) (**Table 3**).

**Table 3:** Recurrence rates early stage endometrioid histology EC by MMR status.

Clinical-pathologic features		MMR proficient	Epigenetic loss *MLH1*	Probable MMR mutation	*p* value
		*N* = 1019 (%)	*N* = 240 (%)	*N* = 68 (%)	
GOG 99 risk classification					
Low or Low intermediate risk					
Recurrence		15 (1.6)	10 (5.8)	1 (1.5)	0.013
No recurrence		896 (98.4)	163 (94.2)	64 (98.5)	
High intermediate risk					
Recurrence		9 (8.3)	9 (13.4)	0	0.410
No recurrence		99 (91.7)	58 (86.6)	3 (100.0)	
PORTEC risk classification					
Low risk					
Recurrence		12 (1.4)	10 (5.7)	1 (1.7)	0.010
No recurrence		822 (98.6)	165 (94.3)	57 (98.3)	
High or High intermediate risk					
Recurrence		12 (6.5)	9 (13.8)	0	0.090
No recurrence		173 (93.5)	56 (86.2)	10 (100.0)	

### Survival

**Figure 1:**
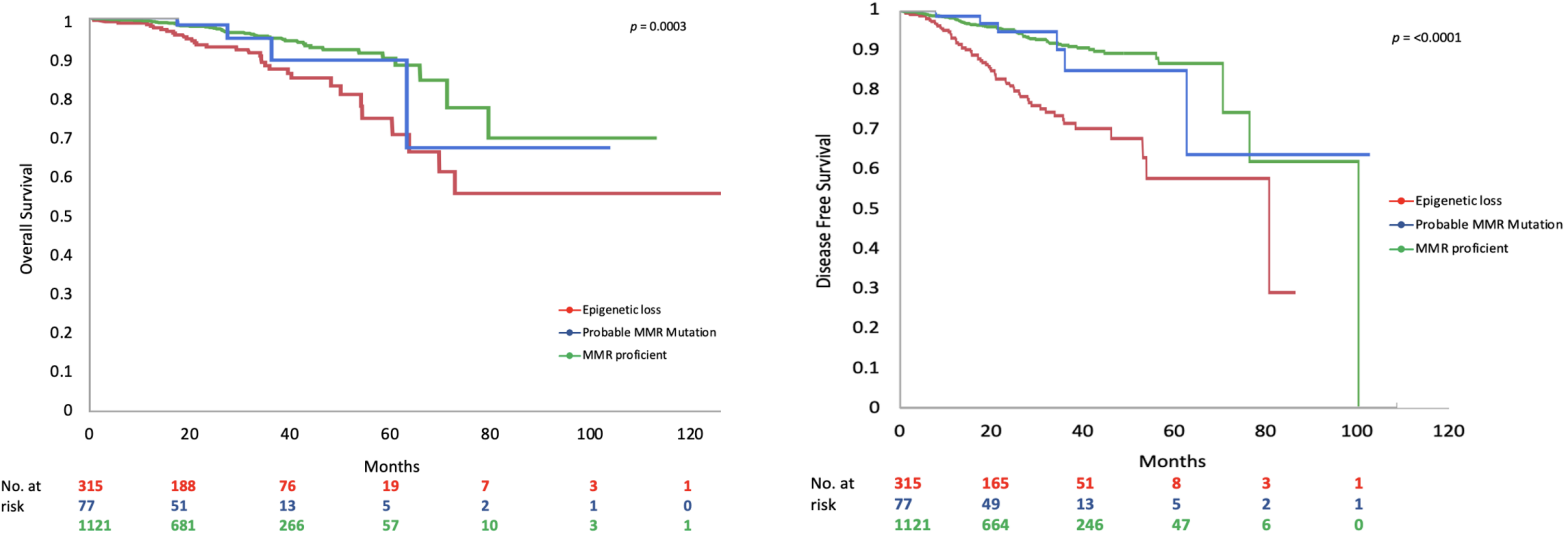
Overall survival and disease-free survival by MMR class.

MMR deficient endometrioid EC had worse DFS (**Figure 1**) and OS than MMR proficient EC (OS data not shown). Only 65.0% of MMR deficient tumors were disease free at 5 years compared to 88.1% of MMR proficient tumors (*p*=**≤**0.001). This detrimental effect appears to be driven by the behavior of EC with epigenetic loss of *MLH1*. The 5-year DFS of EC with epigenetic loss was 57.5% compared to 85% in EC with probable MMR mutation and 88.1% in MMR proficient EC (*p*=**≤**0.001). The 5-year OS for EC with epigenetic loss of MLH1 was 74.6% compared to 89.1% for EC with probable MMR mutation and 90% for MMR proficient EC (*p*=0.003). Univariate analysis revealed that MMR status, age, BMI, stage, grade, and LVSI were significantly associated with survival. When the six factors significant in univariate analysis were included in multivariable analysis (along with adjuvant therapy) MMR status was found to be independently associated with DFS but not OS (HR 1.90; 95% CI 1.34-2.70; *p*=0.003) (**Table 4**).

**Table 4:** Multivariate analysis for endometrioid EC disease-free survival and overall survival.

Variable	Disease-free survival			Overall Survival		
	Hazard ratio	95% CI	*p* value	Hazard ratio	95% CI	*p* value
Age (continuous variable)	1.04	1.02-1.06	**≤**0.001*	1.05	1.02-1.11	**≤**0.001*
BMI (continuous variable)	1.01	0.98-1.03	0.091	0.99	0.98-1.09	0.110
FIGO stage (III/IV vs I/II)	0.39	0.25-0.62	**≤**0.001*	0.59	0.47-0.73	**≤**0.001*
Histologic grade (3 vs 1 & 2)	0.56	0.36-0.89	0.0149	0.99	0.78-1.28	0.991
LVSI (absent vs present)	2.79	1.76-4.42	**≤**0.001*	1.18	0.99-1.40	0.060
Adjuvant therapy (therapy vs no therapy)	0.85	0.53-1.37	0.51	0.89	0.76-1.05	0.172
MMR status (MMR deficient vs MMR proficient)	1.90	1.34-2.70	0.003*	1.03	0.92-1.18	0.540

*Denotes statistical significance. N=1,514 Endometrioid EC, N=85 deaths during follow-up period, N=58 disease related mortality.

In early-stage endometrioid EC, MMR deficiency was associated with significantly worse OS and DFS (**Supplementary Figure S1**). The effect of MMR deficiency on DFS was evaluated *via* Cox proportional hazard ratios; epigenetic loss of *MLH1* was independently associated with worse DFS in early-stage EC after controlling for GOG99 and PORTEC risk classification (HR 2.75; 95% CI 1.69-4.48; *p*=**≤**0.001). Most striking, in patients at low and low-intermediate risk by GOG 99 criteria there was a significantly increased risk for recurrence and worse DFS (5-year DFS 80.8% vs 94.6% at 5 years, *p*=0.004). The effect of MMR deficiency on recurrence risk persisted when evaluating patients at low-risk for recurrence by PORTEC criteria (5-year DFS 70.8% vs 95.4%, *p*=**≤**0.001). Due to the relatively few patients with early-stage EC and probable MMR mutations who recurred (N=3), we are unable to comment on the effect of probable MMR mutation and recurrence risk.

## Discussion

In this study, we confirm and expand on prior reports that epigenetic MMR deficiency in EC is associated with poor prognostic features (9, 24, 38, 39). However, for the first time we demonstrate that MMR deficiency was an independent predictor for lymph node metastasis and recurrence after controlling for these prognostic factors through traditional risk stratification algorithms.

In patients at high risk for LN metastasis by Mayo criteria, EC with epigenetic MMR defect was twice as likely to have LN metastasis (20% vs 10.5%). Tumor size is an established prognostic factor for lymph node involvement and thus has been integrated into risk stratification algorithms used to identify women in whom surgical lymphadenectomy can be safely omitted (23, 25). Indeed, the significantly different rates of lymphadenectomy between MMR deficient tumors (83.7%) and MMR proficient EC (74.1%) illustrates the effect tumor volume may have on surgical decision-making. Our group has previously reported on the association of epigenetic MMR defects with large tumor volume and lymph node metastasis (20) but in this study, we identify that epigenetic MMR defect is associated with lymph node metastasis independent of tumor volume. Recently, Diniz et al. advocated for the use of MMR status to triage patients who require lymphadenectomy (26); however, their analyses did not differentiate between epigenetic loss and those with probable MMR mutation. The association between epigenetic MMR deficits and LN positivity seen in our study suggests that the 22% (15/69) MMR deficient EC with positive LN in their study may largely be attributed to epigenetic MMR deficits.

Factors such as advanced age, higher grade, LVSI, and deeper myometrial invasion are utilized to predict patients at high risk for recurrence despite early stage disease. This study, and others (9, 13, 22, 27, 28), confirms that MMR deficient EC is associated negative prognostic indicators. However, we found that patients with probable MMR mutation did not differ from those with MMR proficient EC in terms of stage, grade, LVSI, and myometrial invasion. Rather, epigenetic MMR defects were the driver for the association of MMR defects with these negative prognostic factors. Given that these prognostic factors are accounted for through risk stratification algorithms (GOG99 and PORTEC) utilized to predict the risk for recurrence and guide adjuvant therapy in early-stage endometrioid EC, we sought to evaluate the role of MMR status after controlling for these factors. We found that there was not a significant difference between MMR proficient EC and EC with probable MMR mutations according to GOG99 and PORTEC risk classification. However, ECs with epigenetic MMR defects were significantly more likely to be classified as high or HIR by traditional risk stratification algorithms; 23.1% vs 10.3% (*p*=**≤**0.001) for GOG99 and 23.4% vs 17.8% (*p*=0.004) for PORTEC criteria. Epigenetic MMR defects were strongly associated with worse DFS in early-stage endometrioid EC independent of risk classification and the receipt of adjuvant therapy.

Finally, when we evaluated all endometrioid ECs, we found that MMR deficiency was independently associated with worse DFS but not OS after controlling for age, BMI, grade, LVSI, stage, and adjuvant therapy.

The association between MMR status and disease recurrence and survival has been extensively studied in a variety of malignancies. Although MMR deficiency has been associated with better prognosis in colorectal cancer its prognostic significance in EC is unclear (29). While many studies have reported worse DFS in patients with MMR deficiency (9, 20, 30, 39) other studies have reported improved outcomes (21, 31, 32, 38). A meta-analysis (33) that sought to evaluate the role of MMR status and clinical outcomes in EC highlights some possible reasons for these discrepancies. Many studies include very small sample sizes (the median sample size in the aforementioned meta-analysis was 112 subjects), heterogeneous patient populations including endometrioid and non-endometrioid histologies, and inconsistent methods for determining and classifying MMR status. Our study has several strengths that we feel empower the findings: (1) The large sample size of more than 1,500 ECs, (2) only endometrioid histology ECs were isolated to avoid histology as a confounding factor, (3) MMR status was classified using the expression of all 4 MMR proteins, and (4) universal *MLH1* methylation testing was performed. Our study does have some important limitations to address including the relatively modest number of recurrences and the very limited number of recurrences in patients with a probable MMR mutation limits the ability to extrapolate the risk profile in this group. In addition, while the median follow-up period of 2.5 years is relatively short, data has shown that the majority of recurrences will occur within 2 years of initial diagnosis.

## Conclusion

Traditional uterine-based risk stratification algorithms may not accurately reflect the risk for lymph node metastasis and recurrence in EC with epigenetic MMR defects. Our findings advocate for the use of molecular classification and MMR testing alongside traditional risk stratification algorithms based on uterine factors. Given the high concordance between MMR IHC status by preoperative biopsy compared to definitive surgical specimen (40–42) these findings also highlight the role of MMR testing on preoperative biopsy specimens to facilitate risk-stratification and patient-centered counseling.

## Data availability statement

The raw data supporting the conclusions of this article will be made available by the authors, without undue reservation.

## Ethics statement

The studies involving human participants were reviewed and approved by The Ohio State University Institutional Review Board. Written informed consent for participation was not required for this study in accordance with the national legislation and the institutional requirements.

## Author contributions

Concept and design (CC, CR, PH). Acquisition, analysis, or interpretation of data (all authors). Drafting of the manuscript (CC, CR). Critical revision of the manuscript for important intellectual content (all authors). Statistical and data analysis (CR, CC). Study supervision (CC, PG, DC). All authors contributed to the article and approved the submitted version.

## Supplementary material

Supplementary Figure 1 OS and DFS in early-stage EC by MMR status.
