# Supplementary figures and images for: Epigenetic MMR defect identifies a risk group not accounted for through traditional risk stratification algorithms in endometrial cancer
Source: Front Oncol. 2023 Apr 6;13:1147657. doi: 10.3389/fonc.2023.1147657 (PMC10117833; doi:10.3389/fonc.2023.1147657)

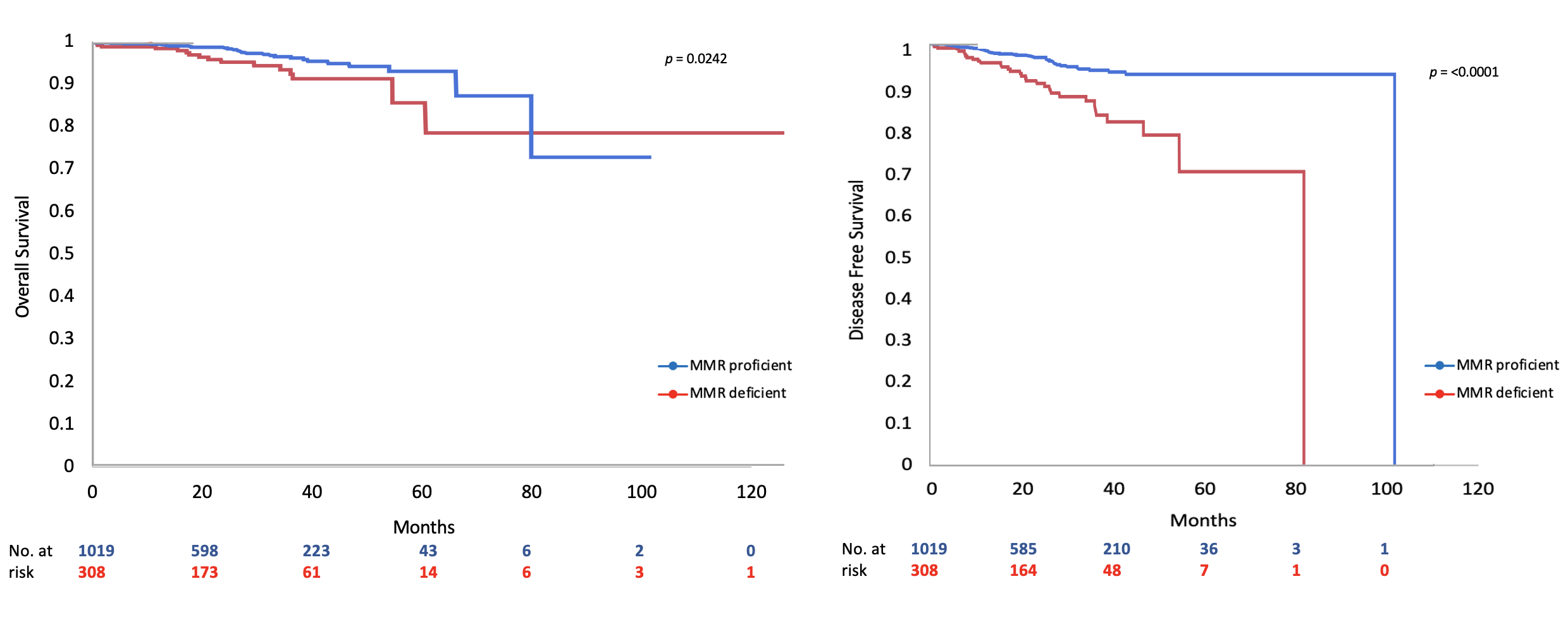

Supplement: Supplementary Figure 1 — OS and DFS in early-stage EC by MMR status. [file Image_1.tiff]
